# Deferrals for Low Haemoglobin and Anaemia Among First-Time Prospective Blood Donors in Southern Ghana: Results From the BLOODSAFE Ghana—Iron and Nutritional Counselling Strategy Pilot (BLIS) Study

**DOI:** 10.1155/ah/9971532

**Published:** 2025-05-06

**Authors:** Catherine Segbefia, Susan Telke, Edeghonghon Olayemi, Caitlin Ward, Lucy Asamoah-Akuoko, Bernard Appiah, Alfred Edwin Yawson, Tara Tancred, Seth Adu-Afarwuah, Amma Benneh-Akwasi Kuma, Michael Ebo Acquah, Solomon Fiifi Ofori-Acquah, Philip Baba Adongo, Reena Ametorwo, Imelda Bates, Cavan Reilly, Yvonne Dei-Adomakoh, BLOODSAFE Ghana Investigators

**Affiliations:** ^1^Department of Child Health, University of Ghana Medical School, Accra, Ghana; ^2^Division of Biostatistics, Coordinating Centers for Biometric Research, University of Minnesota, Minneapolis, Minnesota, USA; ^3^Department of Haematology, University of Ghana Medical School, Accra, Ghana; ^4^Research, Planning Monitoring and Evaluation Department, National Blood Service Ghana, Accra, Ghana; ^5^Department of Public Health, Syracuse University, Syracuse, New York, USA; ^6^Department of Community Health, University of Ghana Medical School, Accra, Ghana; ^7^Department of International Public Health, Liverpool School of Tropical Medicine, Liverpool, UK; ^8^Department of Nutrition and Food Science, University of Ghana, Accra, Ghana; ^9^Department of Haematology, Korle Bu Teaching Hospital, Accra, Ghana; ^10^Department of Medical Laboratory Sciences, School of Biomedical and Allied Health Sciences, University of Ghana, Accra, Ghana; ^11^Department of Social and Behavioural Sciences, School of Public Health, University of Ghana, Accra, Ghana; ^12^The Centre for Capacity Research, Liverpool School of Tropical Medicine, Liverpool, UK

**Keywords:** anaemia, first-time blood donors, Ghana

## Abstract

In Ghana, prevalence of anaemia is higher than the worldwide average and contributes to deferral of blood donors. A cross-sectional study was carried out as part of a pilot study aimed at improving haemoglobin levels and promoting repeat donations to retain donors who were deferred due to low haemoglobin. The copper sulphate test was used to determine low haemoglobin and anaemia assessed by the World Health Organization (WHO) gender-specific criteria. Over sixteen months, 1213 donors were eligible, of which 826 (68%) were male and 78 (6.4%) were deferred for low haemoglobin. Among these 78 deferrals, 71 (91%) were female, 77 (99%) were first-time donors and 77 (99%) were voluntary nonremunerated blood donors (VNRBDs). A total of 337 donors consented to provide a blood specimen out of which 325 donors met eligibility criteria and had complete FBC results. Of those, 189 (*N* = 39 males; *N* = 150 females), or 58%, were classified as anaemic. Model-based estimates which correct for selection bias in the enrolment process found that 61.6% of female donors (95% credible interval: [53.4%, 70.8%]) and 19.7% of male donors (95% credible interval: [11.5%, 33.8%]) were anaemic by WHO criteria. Among the 252 consenting donors with completed blood specimen analyses and haemoglobin levels meeting the threshold for blood donation, 118 (47%) were classified as anaemic according to WHO criteria. Population-level estimates of anaemia using WHO criteria suggest anaemia is highly prevalent and the results generally matched donor deferral using the copper sulphate test among women blood donors.

**Trial Registration:** ClinicalTrials.gov identifier: NCT04949165

## 1. Introduction

In sub-Saharan Africa, there is a large gap between the demand and the supply of whole blood and blood products available for transfusion. Despite a high clinical need for blood, transfusion services and blood banks usually have limited stores. Limited supply of safe blood adversely impacts the delivery of routine and emergency medical services and directly increases mortality risk. Structural aspects of blood donation are believed to play an important role in the lack of safe blood. There are three main types of blood donors: voluntary nonremunerated blood donors (VNRBDs), paid and family replacement donors (FRDs). About two-thirds of blood collection in Ghana is obtained through the FRD system [1], which is considered to be less safe and less reliable compared to a VNRBD system, particularly for emergencies. Regular, unpaid, voluntary donors are vital in ensuring a safe and sustainable blood supply. In countries with a 100% voluntary blood donor base, the average blood donation rate is 31 units per 1000 population. In contrast, the blood donation rate in Ghana saw little increase from 5.8 to 6.0 units per 1000 population between 2016 and 2018, with the proportion of voluntary unpaid donors stagnant at less than 35% over the same period [1]. The low blood donation rate in Ghana is compounded by a high rate of deferrals among potential blood donors. Donors can be temporarily (e.g., anaemia) or permanently (e.g., evidence of hepatitis B) disqualified from donating blood. Donor deferral may occur at any point during the blood donation process.

Anaemia is a modifiable reason for blood donor deferral. The National Blood Service Ghana (NBSG) supports the use of a semi-quantitative gender-specific gravimetric method, called the copper sulphate test to evaluate haemoglobin. When properly performed, deferral thresholds for anaemia are < 13.0 g/dL for males and < 12.0 g/dL for females. In some cases, NBSG sites will perform additional point-of-care haemoglobin analysis using HemoCue. Although the causes of anaemia vary and may be multifactorial, iron deficiency (ID) is the most common and prevalent micronutrient deficiency worldwide [2]. ID is usually due to inadequate dietary intake, further exacerbated by the high burden of malnutrition and parasitic infestations in sub-Saharan Africa [3]. Initially, ID manifests as reduced iron stores and iron-deficient erythropoiesis. Without intervention, ID is complicated by overt anaemia. In blood donors, iron balance is an important safety issue. One complete whole blood donation (400–500 mL) leads to the loss of almost 250 mg of iron. Adolescent and adult females' body iron stores are about 300 mg [4]. A female blood donor, therefore, loses a significant amount of her total body iron and risks becoming iron depleted after just one donation of a unit of whole blood. With continued iron loss from repeat whole blood donations and inadequate replacement, blood donors inevitably develop ID and iron deficiency anaemia (IDA), which result in deferment at subsequent blood donation attempts. In the REDS-II Donor Iron Status Evaluation study in the United States, 15% of blood donors were found to have absent iron stores (serum ferritin < 12 ng/mL), and 41.7% exhibited iron-deficient erythropoiesis (log TfR/ferritin 2.07) [5]. Apart from ID, anaemia can be caused by other micronutrient deficiencies (e.g., folic acid, vitamin B12 and copper), intrinsic red cell defects such as haemoglobinopathies, enzyme deficiencies and membranopathies, abnormal blood loss, acute and chronic infections and other systemic illnesses like cancers, autoimmune disorders and chronic kidney disease.

The global prevalence of anaemia in 2021 was 17.5% (95% uncertainty interval (UI) 17.0–18.0) in males and 31.2% (30.7–31.7) in females [4]. Significant variations have been observed in anaemia burden by age, sex and geographical location, with children younger than five years, women and countries in sub-Saharan Africa and South Asia being the most affected. Prevalence was greatest in western sub-Saharan Africa (47.4% [95% UI 45.1–49.5]) [4]. The prevalence of anaemia in Ghana is higher than the global average, estimated at 42.4% in women of reproductive age [3, 6] and 18.8% among periurban men [6]. This high prevalence of anaemia in the Ghanaian general population may contribute to an increased rate of blood donation deferrals due to low haemoglobin. Deferrals result in a loss of valuable members of the donor pool who could have been retained if they had been identified, counselled and treated appropriately.

Data collected as part of this recruitment effort include recording of NBSG standard data collection from all donors presenting at a site to donate. The use of such standard data did not require study-specific consent. In addition, a substudy of anaemia, ID and IDA, termed the study of ID and IDA, was performed among the subset of first-time VNRBDs who gave consent for additional study-specific data collection including providing a blood specimen at screening. Standard NBSG data collection allows for estimation of the frequency of deferral for low haemoglobin (as assessed by the copper sulphate test) and associated factors among those who would otherwise be able to donate. To address the loss of donors due to anaemia, a pilot study of nutritional counselling and iron supplementation aimed to increase haemoglobin and encourage repeat donation was conducted.

## 2. Methods

### 2.1. Study Design and Sites

The large cross-sectional study on donor deferral, along with the substudy on ID and IDA, formed part of a broader pilot project funded by the National Heart, Lung, and Blood Institute, USA (Grant number 1UG3HL151599-01), titled “Iron Supplementation and Nutritional Counseling Interventions to Improve Availability and Safety of Blood in Ghana” (BLIS). Prospective donors were recruited using the convenience sampling method from three static sites and during mobile drives organised by the Southern Zonal Blood Centre (SZBC) at senior high schools and tertiary institutions within the catchment area. A cross-sectional design was utilised to assess the prevalence of donor deferral due to low haemoglobin in blood donors who passed the initial NBSG prescreening requirements, which included age 17–60 years old, examining for skin lesions/needle marks, passing medical and behavioural history questionnaire and passing vital sign assessment. Low haemoglobin was determined using the copper sulphate method and in some cases with HemoCue 201+ system. HemoCue 201+ system provides a point-of-care haemoglobin value. All participants completed a structured questionnaire to obtain data on age, sex, socioeconomic status (e.g. occupation), medical history (current and past), nutritional status through a nonquantified nutrient food frequency, history of blood loss and use of nonsteroidal anti-inflammatory drugs (e.g., aspirin and ibuprofen). Anaemia was defined according to WHO gender-specific hemoglobin thresholds (haemoglobin ≤12 g/dL for females and ≤13 g/dL for males), using Full Blood Count (FBC) analysis performed with the ABX Micros ES60 OT (Horiba Medical, France). Haemoglobin levels were measured from a subset of donors who provided blood samples in a separate EDTA tube during their donation attempt [7]. ID without anaemia was identified by lower levels of serum ferritin < 15 μg/L. IDA was defined using the WHO gender-specific cutoff values of HB < 12 g/dL for females and HB < 13 g/dL for males and serum ferritin < 15 μg/L.

Nutrition counselling included guidance on consuming everyday foods abundant in iron, like dark green leafy vegetables, as well as incorporating protein-rich foods. Additionally, advice was given to avoid foods that hinder iron absorption, such as tea, coffee, whole grains (which contain phytates) and foods containing tannin. The significance of certain vitamin C-rich foods, such as oranges and pineapples, in enhancing iron absorption was also discussed with the participants.

### 2.2. Statistical Analysis

Baseline characteristics were summarised with counts and percentages for binary and categorical variables and means and standard deviations for continuous variables. Logistic regression was used to examine the impact of sex, age, voluntary donation status and repeat donation status on deferral due to low haemoglobin (as assessed by a copper sulphate or HemoCue test) among all donors. Logistic regression was also used to assess the impact of sex, age and anaemia status from the FBC on deferral for low haemoglobin among those in the ID/IDA study. All hypothesis tests are 2-sided at the 5% significance level.

Due to selection bias during enrolment, where deferred donors were preferentially approached for consent into the study on ID and IDA, a probability model accounting for the nonignorable missing data mechanism was employed to estimate the prevalence of anaemia based on WHO criteria. This model used a logistic regression model which assumed that the probability that someone is anaemic (WHO criteria) among those who did not enrol in the ID/IDA study depends on age, sex and deferral for low haemoglobin (from copper sulphate/HemoCue) in the same manner as it does for those who did enrol. Data from those who did enrol were used to estimate the parameters in this model, and this was used to impute anaemia status (WHO criteria) among those who did not enrol in the study of ID and IDA. This model was combined with sex-stratified probabilities of enrolment. Averaging the estimated and imputed probabilities of anaemia (WHO criteria) by sex across those enrolled and not enrolled yielded sex-specific estimates of the probability of anaemia (WHO criteria) conditional on enrolment status. For both males and females, the final adjusted estimates of the proportion of anaemia (WHO criteria) among donors were obtained by weighting these conditional probabilities by the estimated probability of enrolment. Bayesian inferential methods were used to obtain parameter estimates, and uninformative normal priors with large variances were used on the model coefficients to allow estimation to be influenced primarily by the observed data. Posterior means and 95% credible intervals (CrIs) were used to summarise the posterior distribution. All statistical analyses use R version 3.6.0.

### 2.3. Ethical Considerations

Ethical approval was obtained from the College of Health Sciences, Ethical and Protocol Review Committee, University of Ghana (protocol ID: CHS-Et/M.5-P4.14/2021), and study oversight was provided by a data coordinating centre in Minnesota and also by a data and safety management board set up by NHLBI.

## 3. Results

**Figure 1:**
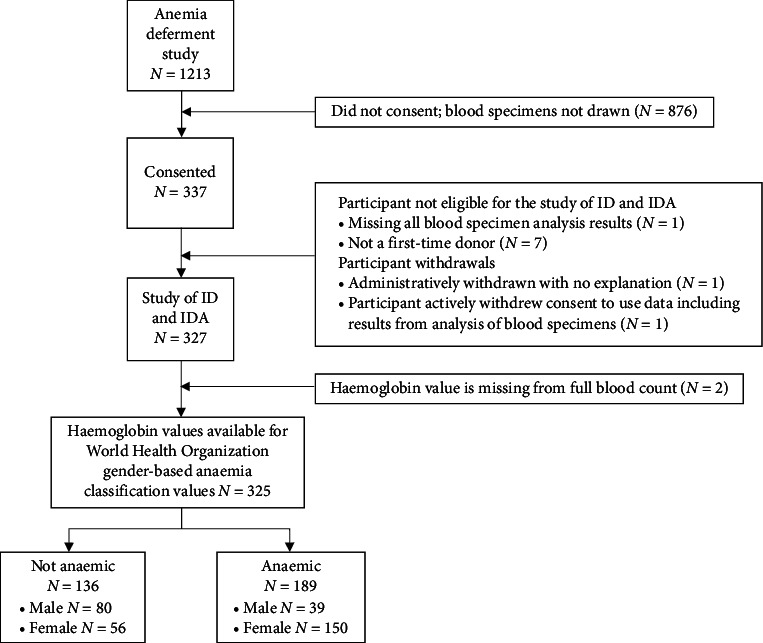
Consort diagram. Consort diagram showing participant recruitment in the substudy of ID/IDA among subset of first-time VNRBDs who gave consent for collection of additional study-specific data and a blood sample. ID = iron deficiency; IDA = iron deficiency anaemia.

The study period was from October 2021 to February 2023. From the blood donation sites, 1213 donors qualified for the study and 337 donors consented to additional data collection which included collection of a blood specimen (Figure 1).

Of the 1213 participants, 387 (32%) were female and 826 (68%) male. The mean age of the participants was 27.3 ± 9.0 years with a range of 17–59 years. Among the females, the average age was 23.2 ± 7.7 years and that of the males was 29.3 ± 9.0 years. Forty-one percent of donors were repeat donors and 37% were VNRBDs. Almost all qualified donors donated successfully, and few adverse events were reported (Table 1).

Of the 1213 participants, 78 (6.4%) were deferred for low haemoglobin by the copper sulphate or HemoCue test. Among these 78 deferrals, 71 (91%) were female, 77 (99%) were first-time donors and 77 (99%) were VNRBDs. The mean age among those deferred was 21 years old compared to a mean age of 28 years old among those not deferred (Table 2).

From the logistic regression model applied to all donors, female donors (aOR = 9.58; 95% CI: 4.52–23.71), first-time donors (aOR = 13.36; 95% CI: 2.09–278.24) and VNRBDs (aOR = 58.07; 95% CI: 10.84–1084.85) had significantly higher adjusted odds of deferral for low haemoglobin (based on outcome of the copper sulphate test or HemoCue result at the time of attempt donation) compared to male, repeat and FRD donors, respectively, when adjusted for age, donor type (repeat/first-time) and type of donation (VNRBD/FRD) (Table 2).

Among 325 participants enrolled in the study of ID/IDA with complete blood specimen results, 189 (58%) donors were classified as anaemic (*N* = 39 males; *N* = 150 females) using the WHO gender-specific haemoglobin values for anaemia (female HB ≤ 12 g/dL; male HB ≤ 13 g/dL) from FBC haemoglobin of blood specimens collected at the donation attempt (Supporting Table 1). Among 73 deferred, consenting first-time donors with completed blood specimen results, 71 (97%) were confirmed to be anaemic (Supporting Table 2). Of the 252 donors who were not deferred, 118 (47%) were found to be anaemic. After adjusting for age and anaemia status, first-time female VNRBDs (aOR = 4.52; 95% CI: 1.92–12.49) had higher odds of deferral for low haemoglobin (from copper sulphate/HemoCue) compared to first-time male VNRBDs. Anaemia status had a large impact (aOR = 28.48; 95% CI: 8.50–177.35) on the probability of deferral for low haemoglobin. Age was not a significant predictor in this model (Table 3).

The missing data model results indicate that among female donors, 61.6% were predicted to be anaemic by WHO criteria (95% CrI: [53.4%, 70.8%]), and among male donors, 19.7% were predicted to be so (95% CrI: [11.5%, 33.8%]).  Table 4 shows the results of the regression model used for imputation.

## 4. Discussion

In this study, we examined the characteristics of donors and the prevalence and risk factors of donor deferral for low haemoglobin. Additionally, we determined the prevalence of anaemia by WHO criteria [7] using data from first-time VNRBDs who consented to additional blood testing. We found that anaemia as defined by WHO criteria is highly prevalent in this population and our estimates are consistent with the published literature. This information is important to guide interventions to treat modifiable causes of donor deferral including low haemoglobin. Reducing donor deferral due to low haemoglobin might retain these valuable members of the donor pool.

Over two-thirds of the study's donors were male, which is consistent with previously published literature in Africa and Asia. However, a review of studies from Europe by Bani and Guissani on gender differences in blood donation showed that apart from Italy and Greece where only one-third of blood donors were female, women comprised approximately 50% of blood donors [8]. The preponderance of male prospective blood donors in this study may be related to low haemoglobin in addition to sociocultural factors, including comparatively lower literacy levels in females, perceptions about males being healthier and misconceptions that women cannot donate blood or that women's main roles should be centred around the household.

Only a little over one-third of this study's donors were VNRBDs, consistent with data from many low- or middle-income countries. The FRD system is considered to place undue burden on patients because blood transfusion or discharge from wards is delayed until friends and/or family can be mobilised to ‘donate' equivalent numbers of units that were transfused. As a result, eligible donors may refrain from voluntary donations in case they are called on to replace blood for relatives. Additionally, because of the lag time in organising FRDs, reliance on replacement donations accounts for inadequate blood for emergencies such as obstetric haemorrhage and severe malaria in children. Moreover, the blood from FRD collections is considered less safe compared to voluntary donations. In the second largest hospital in Ghana, the prevalence of transfusion-transmitted infections was significantly higher in FRD compared to VNRBD (23.5% vs. 3.5%) [9]. The NBSG prohibits the use of paid donors; however, it is unknown if some paid donors pose as friends and family members of patients who need blood. VNRBDs are recommended as the key to countries developing and maintaining safe, accessible and reliable blood supply [10, 11]. Despite the challenges with the FRD system, it is also acknowledged that FRDs are a critical lifeline in the absence of blood from VNRBDs.

In the current study, based on an initial copper sulphate or HemoCue test result, females, first-time donors and VNRBDs were significantly more likely to be deferred. Being female and anaemic (WHO criteria) was associated with significantly higher odds of being deferred. In Ivory Coast, the most common reason for long-term deferral was low haemoglobin level in women [12]. In the United States, being of female gender resulted in 11 times greater odds of being deferred for anaemia, compared to males [13]. Females within the reproductive age group are more likely to be anaemic than males due to menstrual blood loss and pregnancy. Additionally, due to the effect of testosterone, adolescent and adult males have higher baseline haemoglobin levels than females. Reducing the proportion of deferrals in females would require a different haemoglobin cutoff compared to that for males during donor screening and possibly iron supplementation after screening. However, donor safety must remain the paramount consideration. Further screening for contributory causes of anaemia in the deferred donors would also be important including addressing parasitic infestations, poor nutrition and haemoglobinopathies which have been reported in other studies.

Donor deferral for low haemoglobin and anaemia by WHO criteria was highly prevalent among women. The disparities observed in the deferral rates may result from differences in the criteria used to select donors in the various countries, such as Hb levels, age, weight and blood donation interval. Furthermore, it may be because of diverse methods and the differences in haemoglobin cutoff values used to define anaemia.

Providing iron supplementation and nutritional counselling has the potential to result in more qualified female donors, thereby adding to the pool of VNRBDs and the amount of blood available for transfusion.

Our study had some limitations. A major limitation was the selection bias which resulted from prioritising deferred donors for inclusion in the substudy requiring consent. In this regard, it was encouraging that the missing data model provided estimates that are consistent with published estimates. Furthermore, other causes of anaemia were not excluded.

## 5. Conclusion

Donor deferral due to low haemoglobin and anaemia by WHO criteria was highly prevalent among women. Providing iron supplementation and nutritional counselling among donors deferred for low haemoglobin due to ID may result in fewer deferred donors, thereby adding to the available blood supply in Ghana.

## Figures and Tables

**Table 1:** Characteristics of donors^1^.

		Enrolled in the study of ID and IDA		
		Overall *N* (%)	No *N* (%)	Yes *N* (%)
Donors		1213	886 (73.0)	327 (27.0)
Age (years); mean (SD)		27.34 (9.01)	30.10 (8.92)	19.87 (3.05)
Sex	Female	387 (31.9)	179 (46.3)	208 (53.7)
Donor type	First-time	722 (59.5)	395 (54.7)	327 (45.3)
	Repeat	491 (40.5)	491 (100.0)	0 (0.0)
Donation type	VNRBD	454 (37.4)	127 (28.0)	327 (72.0)
	FRD	759 (62.6)	759 (100.0)	0 (0.0)
Copper sulphate test result	Failed	56 (4.6)	3 (5.4)	
	Not done	55 (4.5)	5 (9.1)	50 (90.9)
	Passed	1102 (90.8)	878 (79.7)	224 (20.3)
HemoCue performed	Not done	1068 (88.0)	810 (75.8)	258 (24.2)
	Performed	145 (12.0)	76 (52.4)	69 (47.6)
Donor deferred^2^		78 (6.4)	4 (5.1)	74 (94.9)
Qualified donors (not deferred)		1135	882	253
Successfully donated	No	5 (0.4)	4 (80.0)	1 (20.0)
	Yes	1130 (99.6)	878 (77.7)	252 (22.3)
Reason for donation failure (among unsuccessful donations)				
	Underbled	4 (80.0)	4 (100.0)	0 (0.0)
	Venous access	1 (20.0)	0 (0.0)	1 (100.0)
Adverse event occurred	No	1130 (99.6)	879 (77.8)	251 (22.2)
	Yes	5 (0.4)	3 (60.0)	2 (40.0)
Type of adverse event (among adverse events)				
	Haematoma	1 (20.0)	1 (100.0)	0 (0.0)
	Painful arm	4 (80.0)	2 (50.0)	2 (50.0)

Abbreviations: FRD = family replacement donor; ID = iron deficiency; IDA = iron deficiency anaemia; SD = standard deviation; VNRBD = voluntary nonremunerated blood donor.

^1^Overall percentages are of total number of donors. Percentages for participants enrolled in ID and IDA are within each characteristic.

^2^Deferral for low haemoglobin was based on copper sulphate or HemoCue result at the time of donation.

**Table 2:** Association of donor deferral due to low haemoglobin with age, gender, donation frequency (repeat, first-time) and donation type.

		Overall *N* (%)	Donor deferred^1^		Univariate^2^		Adjusted^3^	
			Yes *N* (%)	No *N* (%)	OR (95% CI)	*p* value	aOR (95% CI)	*p* value
Donors		1213	78 (6.4)	1135 (93.6)				

Age (years); mean (SD)		27.34 (9.01)	20.67 (4.77)	27.80 (9.06)	0.85 (0.80, 0.89)	< 0.001	1.05 (0.99, 1.11)	0.088

Sex	Female	387 (31.9)	71 (18.3)	316 (81.7)	26.3 (12.83, 63.42)	< 0.001	9.58 (4.52, 23.71)	< 0.001
	Male	826 (68.1)	7 (0.8)	819 (99.2)	Ref.		Ref.	

Donor type	First-time	722 (59.5)	77 (10.7)	645 (89.3)	58.5 (12.93, 1033.78)	< 0.001	13.36 (2.09, 278.24)	0.024
	Repeat	491 (40.5)	1 (0.2)	490 (99.8)	Ref.		Ref.	

Donation type	VNRBD	454 (37.4)	77 (17.0)	377 (83.0)	154.82 (34.19, 2736.53)	< 0.001	58.07 (10.84, 1084.85)	< 0.001
	FRD	759 (62.6)	1 (0.1)	758 (99.9)	Ref.		Ref.	

Abbreviations: aOR = adjusted odds ratio; CI = confidence interval; FRD = family replacement donor; OR = odds ratio; SD = standard deviation; VNRBD = voluntary nonremunerated blood donor.

^1^Deferral for low haemoglobin was based on copper sulphate or HemoCue test at the time of donation.

^2^Univariate logistic regression with binary outcome donor deferred.

^3^Logistic regression with binary outcome of donor deferred adjusted for age, sex, repeat donor and donation type.

**Table 3:** Association of donor deferral due to low haemoglobin with age, gender and anaemia status as defined by World Health Organization gender-specific criteria from full blood count haemoglobin of blood specimens collected at donation attempt among donors enrolled in the study of ID and IDA.

		Overall *N* (%)	Donor deferred^3^		Univariate^4^		Adjusted^5^	
			Yes *N* (%)	No *N* (%)	OR (95% CI)	*p* value	aOR (95% CI)	*p* value
Donors^1^		325	73 (22.5)	252 (77.5)				

Age (years); mean (SD)		19.87 (3.05)	20.14 (3.75)	19.79 (2.82)	1.04 (0.95, 1.12)	0.394	1.06 (0.96, 1.17)	0.260

Sex	Female	206 (63.4)	67 (32.5)	139 (67.5)	9.08 (4.10, 24.12)	< 0.001	4.52 (1.92, 12.49)	0.001
	Male	119 (36.6)	6 (5.0)	113 (95.0)	Ref.		Ref.	

Anaemia^2^	Yes	189 (58.2)	71 (37.6)	118 (62.4)	40.31 (12.29, 248.64)	< 0.001	28.48 (8.50, 177.35)	< 0.001
	No	136 (41.8)	2 (1.5)	134 (98.5)	Ref.		Ref.	

Abbreviations: aOR = adjusted odds ratio; CI = confidence interval; ID = iron deficiency; IDA = iron deficiency anaemia; OR = odds ratio; SD = standard deviation.

^1^Haemoglobin blood specimen values available among 327 donors who qualified and consented to the study of ID and IDA.

^2^World Health Organization gender-specific haemoglobin values for anaemia (female HB ≤ 12 g/dL; male HB ≤ 13 g/dL) from full blood count values from blood specimens collected at donation attempt.

^3^Deferral for low haemoglobin was based on copper sulphate or HemoCue test at the time of donation. One consenting donor was deferred and did not have completed screening blood specimen results.

^4^Univariate logistic regression with binary outcome of donor deferral.

^5^Logistic regression with binary outcome of donor deferral adjusted for age, sex and anaemia.

**Table 4:** Posterior means and 95% credible intervals from the Bayesian logistic regression model used for the missing data model predicting anaemia status as defined by World Health Organization among donors enrolled in the study of ID and IDA (*N* = 325).

Model coefficients		OR (95% CrI)
Donor deferral^1^	Deferred	63.96 (9.54, 287.91)
	Not deferred	Ref.
Age (years)		0.92 (0.83, 1.01)
Sex	Female	3.94 (2.26, 6.51)
	Male	Ref.

**Sex-stratified probabilities**		**Mean (95% CrI)**

Enrolment	Female	0.535 (0.484, 0.584)
	Male	0.144 (0.121, 0.169)
Anaemia	Female	0.616 (0.534, 0.708)
	Male	0.197 (0.115, 0.338)

Abbreviations: CrI = credible interval; OR = odds ratio.

^1^Deferral for low haemoglobin was based on copper sulphate or HemoCue test at the time of donation.

## Data Availability

The data that support the findings of this study are available in the Supporting Information of this article.

## Supporting Information

Supporting Table 1: demographics of donors enrolled in the ID and IDA study.

Supporting Table 2: association of full blood count measures with haemoglobin and ferritin.
