# Supplementary material for: Deferrals for Low Haemoglobin and Anaemia Among First-Time Prospective Blood Donors in Southern Ghana: Results From the BLOODSAFE Ghana—Iron and Nutritional Counselling Strategy Pilot (BLIS) Study
Source: Adv Hematol. 2025 May 6;2025:9971532. doi: 10.1155/ah/9971532 (PMC12074844; doi:10.1155/ah/9971532)
Supplement: Supporting Information — Additional supporting information can be found online in the Supporting Information section. [file 9971532.f1.docx]

**SUPPLEMENTAL TABLES**

Supplemental Table 1. Demographics of donors enrolled in the ID and IDA study.

|  |  | Overall  N (%) |
| --- | --- | --- |
| Donors enrolled^1^ |  | 327 |
| Marital status | Single | 320 (97.9) |
|  | Married | 6 (1.8) |
|  | Cohabiting | 1 (0.3) |
| Children | No | 323 (98.8) |
|  | Yes | 4 (1.2) |
| Number of children^2^; mean (SD) |  | 1.75 (0.96) |
| Home situation | I live alone | 25 (7.6) |
|  | I live with only my husband/wife/partner | 2 (0.6) |
|  | I live with my husband/wife/partner  and children | 4 (1.2) |
|  | I live with my parents/relatives/friends | 283 (86.5) |
|  | Other | 13 (4.0) |
| Main method of travel | Private | 18 (5.5) |
|  | Public | 309 (94.5) |
| Highest level of education completed | None | 1 (0.3) |
|  | Junior high school | 220 (67.3) |
|  | Senior high school | 95 (29.1) |
|  | Diploma | 3 (0.9) |
|  | Degree | 7 (2.1) |
|  | Postgraduate level | 1 (0.3) |
| Employment categories | Student | 318 (97.2) |
|  | Informal/Part-time employment | 2 (0.6) |
|  | Self-employed | 1 (0.3) |
|  | Formal employment | 6 (1.8) |
| Ethnic background | Akan | 160 (48.9) |
|  | Ewe | 60 (18.3) |
|  | Dagbani | 3 (0.9) |
|  | Ga/Dangbe | 72 (22.0) |
|  | Hausa | 7 (2.1) |
|  | Other | 25 (7.6) |
| Belong to a religious faith | Christian | 305 (93.3) |
|  | Muslim | 22 (6.7) |
| Owns | Car | 16 (4.9) |
|  | Motorbike | 10 (3.1) |
|  | Bicycle | 25 (7.6) |
|  | Television | 138 (42.2) |
|  | Refrigerator or deep freezer | 128 (39.1) |
|  | Radio | 121 (37.0) |
|  | Cooking stove | 145 (44.3) |
|  | None of the listed items | 126 (38.5) |

ID = Iron Deficiency; IDA = Iron Deficient Anaemia; SD = Standard Deviation

^1^One ID and IDA participant withdrew consent to use screening data.

^2^Among donors with at least one child

Supplemental Table 2. Association of full blood count measures with haemoglobin and ferritin.

|  |  | Sex | | Haemoglobin^3^ | | Ferritin^4^ | |
| --- | --- | --- | --- | --- | --- | --- | --- |
|  | Overall  Average (SD) | Female Average (SD) | Male  Average (SD) | *β* | p-value | *β* | p-value |
| Donors^1^ | 325 | 206 (63.4) | 119 (36.6) |  |  |  |  |
| Haemoglobin (g/dL) | 12.09 (1.77) | 11.23 (1.34) | 13.57 (1.43) |  |  | 0.3 | <0.001 |
| Log (ferritin + 1) (u/L) | 3.78 (0.94) | 3.44 (0.84) | 4.38 (0.80) | 0.7 | <0.001 |  |  |
| WBC (10^9^/L) | 5.80 (1.61) | 5.97 (1.69) | 5.51 (1.43) | 0.1 | 0.004 | 0.0 | 0.583 |
| Lymph (10^9^/L) | 2.77 (0.82) | 2.81 (0.83) | 2.71 (0.79) | 0.3 | 0.001 | 0.1 | 0.255 |
| Mono (10^9^/L) | 0.37 (0.18) | 0.38 (0.20) | 0.35 (0.15) | 1.5 | <0.001 | 0.3 | 0.330 |
| GRA (10^9^/L) | 2.67 (1.12) | 2.79 (1.17) | 2.46 (0.99) | 0.1 | 0.224 | -0.0 | 0.846 |
| MCV (fL) | 83.35 (7.65) | 83.26 (8.01) | 83.51 (7.00) | 0.1 | <0.001 | 0.0 | <0.001 |
| MCH (pg) | 26.76 (3.04) | 26.79 (3.09) | 26.72 (2.96) | 0.2 | <0.001 | 0.1 | <0.001 |
| RDW (%) | 16.25 (1.61) | 16.39 (1.74) | 16.01 (1.35) | -0.4 | <0.001 | -0.3 | <0.001 |
| PLT (10^9^/L) | 235.50 (66.57) | 248.31 (67.00) | 213.26 (59.86) | -0.0 | <0.001 | -0.0 | <0.001 |
| Abnormal Peripheral Blood Film;^2^ N (%) | 198 (60.9) | 153 (77.3) | 45 (22.7) | -1.6 | <0.001 | -0.5 | <0.001 |
| Lymphocytosis | 15 (4.6) | 12 (80.0) | 3 (20.0) | 0.0 | 0.903 | -0.1 | 0.736 |

ID = Iron Deficiency; IDA = Iron Deficient Anaemia; SD = Standard Deviation.

^1^Among donors enrolled in the ID and IDA study with screening laboratory values are available.

^2^No participants had evidence of eosinophilia, malaria, neutrophilia or leukocytosis from peripheral blood film analyses.

^3^Linear regression with outcome of haemoglobin adjusted for age and sex.

^4^Linear regression with outcome of log (ferritin + 1) adjusted for age and sex.
